# A Man Living in the Wilderness Presents With a Unique Case of *Erysipelothrix rhusiopathiae* Causing Primary CNS Infection

**DOI:** 10.1155/crdi/6625621

**Published:** 2025-03-19

**Authors:** Kevin Andrew Smith, Alejandra Méndez, Lindsey J. Reese

**Affiliations:** ^1^Infectious Disease, Indiana University School of Medicine, Indianapolis, Indiana, USA; ^2^Internal Medicine, West Virginia University School of Medicine, Morgantown, West Virginia, USA

**Keywords:** central nervous system, clinical management, craniectomy, *Erysipelothrix rhusiopathiae*, subdural hematoma

## Abstract

We report the first published case of *Erysipelothrix rhusiopathiae* causing subdural empyema. This 66-year-old male patient had relevant exposure history including living in a tent in the woods and having direct contact with wildlife. His main symptom which triggered his presentation was diplopia with exam findings consistent with a left partial oculomotor nerve palsy. Initial attempts at less invasive source control via burr holes alone failed. He was treated successfully with craniectomy and aqueous penicillin G with a duration of 6 weeks following surgery. CNS infection as the primary manifestation of *Erysipelothrix rhusiopathiae* has been reported in only one other case based on our review of the literature. This pathogen demonstrates an ability to manifest infection in many ways and remains susceptible to narrow spectrum beta-lactams.

## 1. Introduction

*Erysipelothrix rhusiopathiae* (formerly *E. insidiosa*) is a nonsporulating, facultative, Gram-positive bacillus. It is ubiquitous and has been found to be commensal in many species of mammals, poultry, and fish, including livestock and domesticated animals. Thus, occupational exposure, such as farming, butchering, fish mongering, and veterinary medicine, is a major risk factor for contact-based infection [1, 2]. Human infection by this bacterium is infrequent and typically presents in one of three forms: (1) erysipeloid (local skin infection), (2) generalized skin infection, and (3) bacteremia with or without endocarditis [2]. It has rarely been identified as a cause of infection in the central nervous system (CNS), and on review of current literature, we are reporting the first published case of *E. rhusiopathiae* as an infectious source of subdural empyema [3–5].

## 2. Case Report

A 66-year-old male with a history of alcohol use disorder and hypertension presented to our emergency department. He endorsed frontal and left-sided headache, gait instability, and diplopia progressing over 1 month. He reported having a fall in a bathtub 7 months earlier, resulting in a concussion and another fall 2 days prior to admission. He lived in a tent in the wilderness and had frequent contact with a feral cat, racoons, and opossums.

On presentation, he was found to have a temperature of 37.1°C, heart rate of 84 beats per minute, blood pressure of 184/109 mmHg, respiratory rate of 14 breaths per minute, and oxygen saturation of 99%. On physical examination the patient appeared poorly groomed and had linear excoriations on the bilateral extensor forearms and legs, consistent with animal scratches which he confirmed were from his outdoor cat. He, otherwise, had a normal exam except for the neurological exam. At rest, the patient demonstrated horizontal gaze deviation. When testing rightward gaze, his diplopia worsened with incomplete adduction of the left eye. On leftward gaze, the diplopia improved with normal abduction of the left eye. The pupils were equal bilaterally. On gait testing, posture was normal but the patient was unsteady and ambulated slowly. Other cranial nerve testing was normal. Finger to nose, heel to shin, and rapid alternating movement tests were normal. His strength, reflexes, and gross sensation were all normal as well.

A CT scan of the head was performed to evaluate the cause of the patient's headache in the setting of falls and cranial nerve palsy. It was remarkable for acute on chronic left subdural hemorrhage with maximal thickness of 1.7 cm with a 5 mm rightward midline shift. The patient underwent middle meningeal artery (MMA) embolization with improvement of symptoms and was discharged home. Three days after discharge, the patient returned with a worsening headache. A repeat CT scan showed a similar sized subdural hemorrhage with no acute bleeding. To reduce intracranial pressure, neurosurgery created burr holes and collected cultures of the bloody fluid to assess for superinfection. The patient reported improved symptoms and was discharged home the following day.

Anaerobic culture of the fluid was found to have *E. rhusiopathiae*, identified using matrix-assisted laser desorption ionization time-of-flight mass spectrometry (MALDI-TOF MS). The gram stain showed no organisms with rare (1+) leukocytes. No other microbes were identified. The patient was initially started on ceftriaxone 2 g every 12 h and antibiotic susceptibility testing demonstrated a minimum inhibitory concentration (MIC) value in mg/L of ≤ 0.03 for penicillin, ≤ 0.06 for ampicillin, ≤ 0.25 for ceftriaxone, 0.25 for erythromycin, 0.12 for clindamycin, and ≤ 0.25 for levofloxacin. Two sets of blood cultures had no growth.

**Figure 1:**
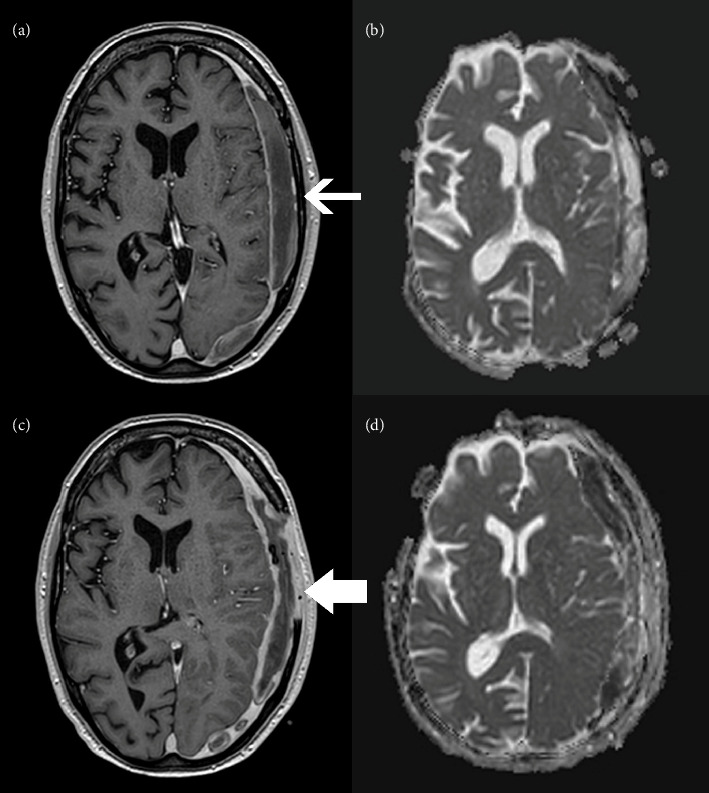
(a) Image from axial view MRI demonstrating mild rim enhancement (narrow arrow) and (b) diffusion restriction after burr hole surgery. (c) Repeat MRI images of similar axial views now demonstrating more marked rim enhancement (wide arrow) and (d) diffusion restriction following craniectomy. These findings are consistent with subdural empyema.

The patient underwent MRI of the head with contrast to evaluate for focal infection (Figures 1(a) and 1(b)). The images demonstrated a biconvex crescent with rim enhancement and restricted diffusion along the left cerebral convexity, consistent with a subdural empyema. There were no other signs of focal infection such as abscess, sinusitis, and mastoiditis.

The patient's hospital course was prolonged by delayed improvement of headache and diplopia. Source control was found to be insufficient with aspiration, burr holes, or drain placement. A craniectomy was ultimately performed with removal of a portion of the skull to eradicate the infection. At the time of this surgery, repeat routine, AFB, and fungal cultures were collected and the fluid collection was evacuated. Despite having completed 10 days of ceftriaxone prior to undergoing craniectomy, anaerobic cultures remained positive for *E. rhusiopathiae.* There were also now many (4+) leukocytes on staining of cultured fluid. He was diagnosed with subdural empyema based on these cultures and the intraoperative signs of inflammation including purulence. He was placed on combined ceftriaxone and continuous aqueous penicillin G for 5 days and then narrowed to penicillin alone.

He returned to the operating room 11 days later for a repeat evacuation due to worsening headache and unresolving diplopia. A purulent collection was identified intraoperatively that had not been completely drained during initial surgery. Repeat cultures of the empyema were collected during this procedure and were negative. A second MRI was also performed after this surgery due to his persistent headache. This revealed increased rim enhancement and diffusion restriction (Figures 1(c) and 1(d)), but due to clinical stability, these were felt to represent expected evolution of his disease process and postoperative changes, so no new interventions were performed. He also had a transthoracic echocardiogram that did not show evidence of endocarditis. The patient was scheduled to undergo cranioplasty in 3 months pending complete resolution of the infection.

The patient completed six more weeks of aqueous penicillin from the time of this procedure. He had prolonged but steady improvement in symptoms with diplopia as the only remaining symptom at 2-month follow-up. Repeat MRI after completion of antibiotics showed a continued rightward midline shift but no new or acute findings.

## 3. Discussion

In general, *E. rhusiopathiae* is transmitted by contact most often from animals and occasionally directly from soil [1, 2]. We hypothesize that our patient was exposed via cutaneous inoculation by excoriation from an outdoor animal exposure. An additional possibility is direct transmission from the soil as he spent much of his time in the wilderness.

Our patient likely developed a subdural hematoma following one of his falls. From this hematoma, he developed a partial left oculomotor nerve palsy, which led him to present to the emergency department. Most likely, this hematoma was seeded with *E. rhusiopathiae* during transient bacteremia or an unrecognized episode of erysipeloid originating from an environmental exposure. There was a large window of time for this to occur as the patient was mildly symptomatic for nearly a month prior to initial presentation. In addition, he first underwent noninvasive MMA embolization, went home, and then returned with continued symptoms before having his first culture from the hematoma over a week after his first presentation. His microbiologic diagnosis was made using MALDI-TOF MS. Due to advances in microbiology and identification, encounters with lesser-known pathogens are becoming more frequent, as in this patient's case [6].

There is one additional reported case of *E. rhusiopathiae* infection associated with the CNS that does not report concomitant endocarditis [3]. In this case, the patient was a farmer who developed erysipeloid and 2 months later developed epidural abscesses and spondylitis. A thorough review of the literature identified no cases of spontaneous CNS infections. Given the mild nature of erysipeloid, it remains a strong possibility that our patient had this skin infection first, although he denied edematous or erythematous plaques at any point in the year prior to this empyema.

Infective endocarditis warrants some consideration in this patient given the known association of *E. rhusiopathiae* and infective endocarditis as well as a published case of meningitis believed to have developed from underlying endocarditis [4]. A key distinction between our case and the case published by Jeong Joo et al. is the presence of fever and constitutional symptoms. Indeed, at presentation, our patient had no fevers (even across three separate admissions) and no other exam findings or lab abnormalities that are specific for endocarditis. In addition, his blood cultures never grew the organism.

A challenge was presented by the lack of published MIC breakpoints for *E. rhusiopathiae* from common governing bodies such as the clinical and laboratory standards institute (CLSI) or the European Committee on Antimicrobial Susceptibility Testing (EUCAST). Instead, we extrapolated susceptibility based on experience with other Gram-positive organisms such as *Streptococcus pneumoniae*, which has an MIC breakpoint of 0.06 mg/L. We initially proceeded with combination therapy for 5 days with ceftriaxone and aqueous penicillin G to allow time for the patient's repeat cultures to finalize in the event of an unidentified polymicrobial infection.

A final important point demonstrated by this case is the exquisite susceptibility of this pathogen to antibiotics, most notably the beta-lactams. This presented an unexpected challenge for our microbiology lab as the amount of dilution necessary to determine MIC was outside of routine protocol which caused some delay. Our findings were consistent with published data on *E. rhusiopathiae* in which 10 isolates were tested for susceptibility [7]. In this publication, it was very sensitive to penicillin. In addition to having a very narrow spectrum, penicillin also has adequate CNS penetration, specifically aqueous penicillin G [8]. For these reasons, we chose to treat him with intravenous aqueous penicillin G. Due to concerns about maintaining an intravenous catheter while living in a tent, the decision was made to admit him to a nursing facility until treatment was completed. The patient's duration of treatment of 6 weeks was based on clinical improvement and expert guidance. There is a lack of clinical trials investigating treatment duration for entities such as subdural empyema, epidural abscess, and pyogenic brain abscess. Expert consensus documents recommend a minimum treatment duration of 4 weeks [9, 10].

To conclude, we report to our knowledge the first published case of *E. rhusiopathiae* causing a subdural empyema in a patient with extensive soil and animal exposure. The challenges of navigating this case in light of the limited published experience were fully described and included evaluation for endocarditis, antibiotic selection, and treatment duration. Definitive resolution was achieved with surgical drainage and long-term intravenous penicillin therapy. The patient has experienced near-complete recovery of neurologic function and remains free of infection.

## Data Availability

Data sharing not applicable to this article as no datasets were generated or analyzed during the current study.
